# Improving the structural, optical, and electrical properties of carboxymethyl cellulose/starch/selenium oxide nanocomposites for flexible electronic devices

**DOI:** 10.1038/s41598-024-53268-w

**Published:** 2024-02-10

**Authors:** Adel M. El Sayed, Tarek I. Alanazi

**Affiliations:** 1 Physics Department, Faculty of Science, Fayoum University, El-Fayoum, 63514 Egypt; 2 Department of Physics, College of Science, Northern Border University, 73222 Arar, Saudi Arabia

**Keywords:** Biomaterials, Condensed-matter physics, Materials for optics, Nanoscale materials, Structural materials

## Abstract

Nanocomposites based on biopolymers are interesting materials owing to their multifunctionality and ease of preparation. In this study, the solution casting method was used to mix selenium oxide nanoparticles (SeO_2_ NP) made by a solvothermal method into a bio-blend of carboxymethyl cellulose and starch (CMC/St). XRD analysis showed that SeO_2_ NP increased the amorphous portion inside the blend. HR-TEM revealed the spherical morphology of these NP with an average diameter of 16.88 nm. The FE-SEM indicated a satisfactory uniform distribution and homogeneity in the surface morphology of the films. FTIR confirmed the interaction between SeO_2_ and the blend functional groups. The films preserved good transmission after doping, and their direct and indirect band gaps decreased. The refractive index, absorption index, optical conductivity, and other dispersion parameters were improved after SeO_2_ loading. The DC conductivity of the blend is in the range of 3.8 × 10^−7^ to 5.6 × 10^−4^ S/m and improved after loading SeO_2_ NP. The IV characteristic curves in the temperature range of 300–415 K were studied to figure out the conduction mechanism in the CMC/St/SeO_2_ composites. Because the optical and electrical properties improved, these nanocomposites could be used for coatings and other things like waveguides, photovoltaic cells, and light-emitting diodes.

## Introduction

The increasing need for eco-friendly multifunctional polymeric materials arises from some concerns related to environmental preservation and technological progress. Combining two or more biocompatible polymers in a blend that combines the advantages of the individual polymers can partially satisfy these requirements. The blending approach yields novel materials that could be used in a wider range of products and applications, such as UV absorbers, sensors, membranes, radiation shields, microelectronic devices, food packaging, and drug delivery systems. The properties of the blend can also be improved by adding nano-sized materials to the blend matrix. This is called polymeric nanocomposite formation^1–4^. The chitin/cashew gum blend had better thermal and mechanical properties than the single polymers. Adding 10% wt% Fe_3_O_4_ NP to the mix made it ideal for magnetic shielding materials and actuators^5^. Adding nanochitosan to polyvinyl alcohol (PVA) and cashew gum improved the semiconducting and dielectric properties of the blend and made it suitable for capacitors and flexible energy storage applications^6^. A nanocomposite made of carboxymethyl chitosan/cashew gum matrix loaded with boehmite NP was used by Meera and Ramesan^7^ for charge harvesting devices and flexible electronics. The material at the nanoscale displays unique physicochemical features in comparison with its bulk counterparts. This is owing to the high surface area and quantum confinement effects^8,9^.

Two of the most easily obtained polymers from renewable resources are cellulose and its CMC derivative, known as CMC or sodium salt. CMC is hydrophilic, semi-crystalline, has good film-forming ability and physiological inactivity, is biodegradable, and is nontoxic. Besides, it has good thickening, viscosity, stabilizing, and binding abilities and can produce flexible and strong films by blending with proteins^2,10,11^. These features make CMC suitable for various uses in medicine, pharmacy, agriculture, industry, and several products such as lotions, creams, and toothpaste. However, despite these advantages, some limitations to using CMC alone exist. Blending CMC with other polymers can mitigate these problems. Salem et al.^2^ found that adding polypyrrole enhanced the thermal stability of CMC but achieved optical conductivity on only 10^11^ S^−1^. Another biodegradable, abundant, and non-toxic polymer is starch (St). This material consists of linear amylose and amylopectin that are clustered and branched^12^. Like CMC, it is widely used for food and non-food utilization, including paper and textiles, wound dressings, and drug carriers. Cheap CMC/St hydrogels with high water uptake were prepared by gamma irradiation for superabsorbent polyelectrolytes^13^. According to El Miri et al.^14^, adding cellulose nanocrystals (0.5–5.0 wt%) to the CMC/St blend improved its viscoelastic and mechanical properties. The bio-nanocomposites that were made are good for use in packaging. There was research by Sun et al.^15^ that looked at how sodium alginate, starch, xanthan gum, gelatin, propanetriol, sorbitol, PEG400, and PEG 6000 changed the thermal and mechanical properties of carboxymethyl bacterial cellulose. A suitable thermal stability was achieved when 1.0% sodium alginate and 0.2% propanetriol were added.

SeO_2_ is a photosensitive semiconductor that is a promising material for photosensors, photovoltaics, and photoelectrochemical applications^16,17^. The solvothermally prepared SeO_2_ nanorods were used as a nanoadditive and a catalyst and were found to be able to make the diesel oil hotter and more volatile^18^. On the other hand, a selenium-free diet leads to liver malfunction and immune and neural system dysfunction^19^. The daily intake of Se for humans should be in the range of 60–70 mg. Some bacteria can’t form biofilms on SeO_2_ NP^20^. It can also kill microbes and cancer cells and break down the methylene blue dye when exposed to visible light. Therefore, SeO_2_ is useful for water treatment, antifungal drugs, and other interesting fields^21,22^.

A few reports on the preparation, optical, and AC electrical properties of polymer/SeO_2_ are found in the literature. Some researchers^1,3^ studied the antibacterial activity of a SeO_2_-doped PVP/CMC blend. They also found that adding 0.8 wt% SeO_2_ made the blend more conductive, going from 6.45 × 10^−7^ S/cm to 2.24 × 10^−6^ S/cm at room temperature (RT)^3^. The effect of SeO_2_ NP and Se/Ag amounts on the optical and dielectric properties of chitosan/polyacrylamide (PAAm) was also reported^16,23,24^. To the best of the author’s knowledge, no report on CMC/St/SeO_2_ bio-nanocomposites has been found. This work is devoted to leveraging the physicochemical properties of CMC, St and nanosized SeO_2_ and studying the structure, morphology, and optical properties of the prepared CMC/St/SeO_2_ nanocomposites. Moreover, the IV characteristic, DC conductivity, and conduction mechanism are discussed.

## Experimental section

### Chemicals and preparation

Selenium (Se) powder and NaBH_4_ (sodium borohydride) as a capping agent were supplied by Sigma Aldrich. Starch powder of molecular weight 260,000 g/mol and chemical formula (C_6_H_10_O_5_)_n_, was supplied by Advent Chembio Pvt. Ltd (India). CMC powder (C_8_H_15_NaO_8_)_n_ of molecular weight 90,000 g/mol, was supplied by El-Nasr Co., (Egypt). Double-distilled (DD) water was used as a common solvent. 0.1 M of Se and 7.5 M of NaBH_4_ were dissolved in 35 mL DD water using a magnetic stirrer at RT. The color of Se turned into a black precipitate after 2 h of stirring. Then the solution was transferred into an autoclave (100 mL) and processed at 175 °C for one day. The precipitate (selenium hydroxides) was then filtered and washed five times with DD water, ethanol, and methanol. Finally, the powder was heat-dried at 100–110 °C in an air furnace and annealed at 400 °C in an Ar atmosphere for 2 h. CMC/St (90%/10%) blend solutions were prepared by dissolving 0.9 g CMC in 40 ml DD water by stirring for 2.0 h at 85 °C and then dissolved in 0.1 g St/10 mL DD water, and the stirring continued for 1.0 h. Using magnetic stirring and ultrasonication, composite (CMC/St/SeO_2_) solutions were prepared by adding the required amount of SeO_2_ NP inside the blend solution. The CMC/St blend and CMC/St/SeO_2_ solutions were cast in cleaned glass Petri dishes and dried slowly at 35–40 °C for several days.

### Characterization and measurements

The PANa-lytical’s X’Pert PRO X-ray diffractometer was operated to record XRD patterns of SeO_2_ powder, CMC/St, and CMC/St/SeO_2_ films. This is done using the Cu-*K*_α_ radiation of wavelength 1.54 Ǻ, 30 kV, and 30 mA, and in the 2θ range of 5°–80°. A high-resolution transmission electron microscope (TEM) (JEM 2100/Jeol/Japan) was used to look at the shape and size of the SeO_2_ powder particles. The surface morphology of the films combined with their thickness through the cross-sectional investigation was analyzed using field emission scanning electron microscopy (FE-SEM) coupled with EDAX, supplied by Carl ZEISS Sigma 500 VP. Fourier transform infrared (FTIR)/attenuated total reflection (ATR) analysis was performed in the range 4000–400 cm^−1^ with a VERTEX 70/70v spectrometer from Bruker Corporation, Germany, and Platinum Diamond ATR. The UV–vis-NIR transmittance and reflectance spectra, in the range of *λ* = 200–1600 nm, were obtained using a JASCO 630 spectrophotometer. All these measurements were carried out RT. A Keithley 2635A computerized system was used to measure the current–voltage (I–V) characteristics of the CMC/St blend and the CMC/St/SeO_2_ nanocomposite films. The temperature range was 300–415 K, and the voltage range was 0.5–20 V. Two Al electrodes were used on both surfaces of the films to make good contact.

## Results and discussion

### Structural and morphological investigation

**Figure 1:**
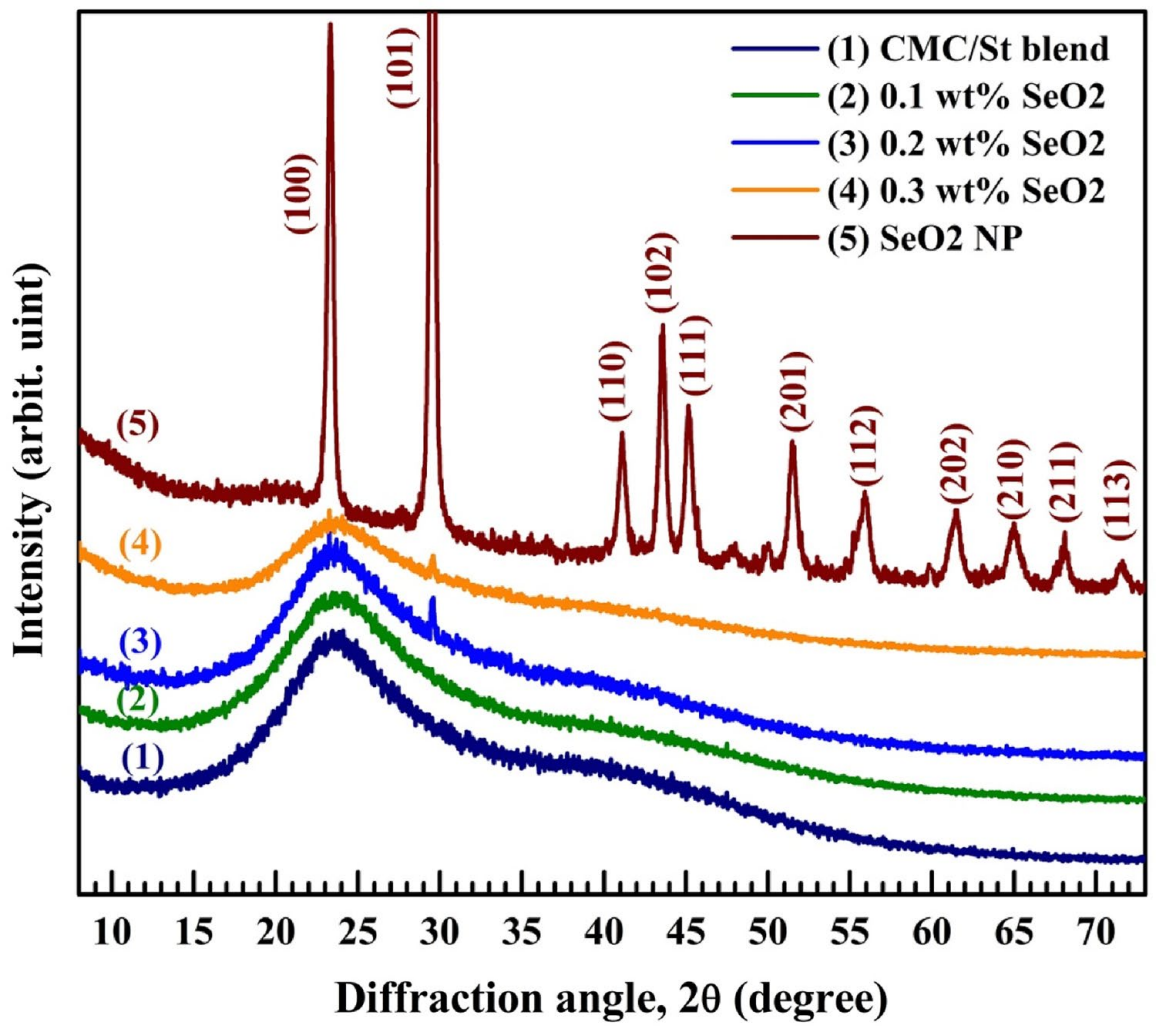
XRD patterns of CMC/St, CMC/St/SeO_2_ nanocomposites and SeO_2_ NP.

XRD patterns of CMC/St, CMC/St/SeO_2_ films, and SeO_2_ powder are presented in Fig. 1. The pure blend and nanocomposite films exhibit a broad peak around 2θ = 23.5°. This peak confirms the semicrystallinity of the blend. It was reported that the XRD pattern of CMC has a wide and broad peak centered at 2θ = 20.7°^25^. The observed shift of the main peak of the blend to 2θ = 23.5° suggests the existence of interactions and intermolecular hydrogen bonding among the chains of CMC and St^8^. Salem et al.^2^ also found a broad peak centered at 2θ = 22.13° in XRD patterns of CMC/polypyrrole. In addition, the observed decrease in the peak intensity, *i.e.*, the peak became broader, after loading 0.1–0.3 wt% SeO_2_ NP indicates the increase of the amorphous regions in the nanocomposite films. The decrease in film crystallinity, as a result of the interactions between CMC/St chains and the SeO_2_, means more flexibility and improved optical and electrical properties, as will be discussed in the following sections.

The XRD pattern of SeO_2_ NP is also shown in Fig. 1. The peaks seen at 2θ = 23.38°, 29.39°, 41.19°, 43.51°, 45.49°, 51.51°, 55.79°, 61.39°, 65.05°, 67.96°, 71.41°, and 76.79° are assigned to the (100), (101), (110), (102), (111), (201), (112), (202), (210), (211), (113), and (212) diffraction planes, respectively. These peaks or planes match well with those of the crystalline SeO_2_, consistent with JCPDS card no. 06–0362^26^. A similar finding was reported for SeO_2_ spherical NP prepared from sodium selenite and some plant leaves^24^. The crystallite size ($D$) of these particles was determined using the well-known Scherrer’s equation and found to be 15.9 nm. XRD patterns of the blend loaded with 0.2 and 0.3 wt% SeO_2_ contain a small peak at 29.39°, which belongs to the most intense (101) crystalline plane of SeO_2_ NP. Other planes disappeared in amorphous regions of the CMC/ST blend and were not detected due to the detection limit of the XRD technique. This result confirms the successful incorporation of SeO_2_ NP inside CMC/St blend chains.

**Figure 2:**
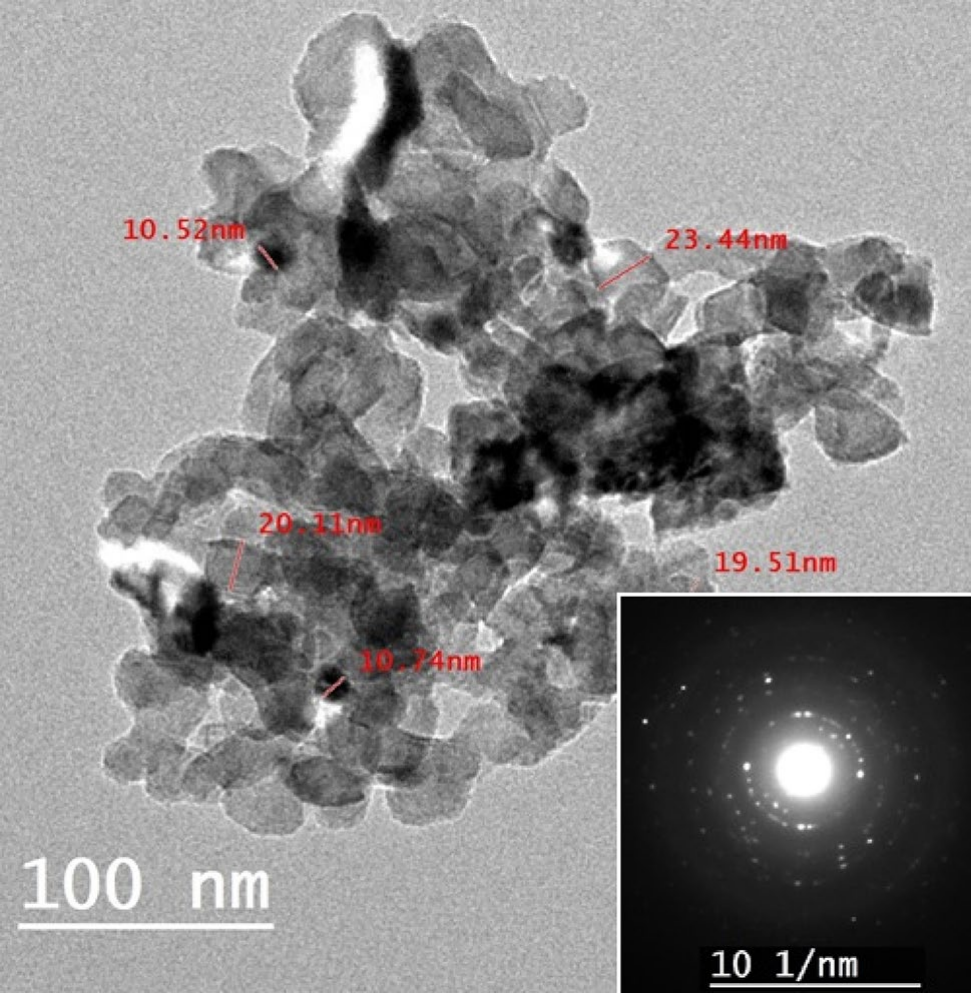
HR-TEM image for SeO_2_ NP. The inset is the SAED for a single particle.

**Figure 3:**
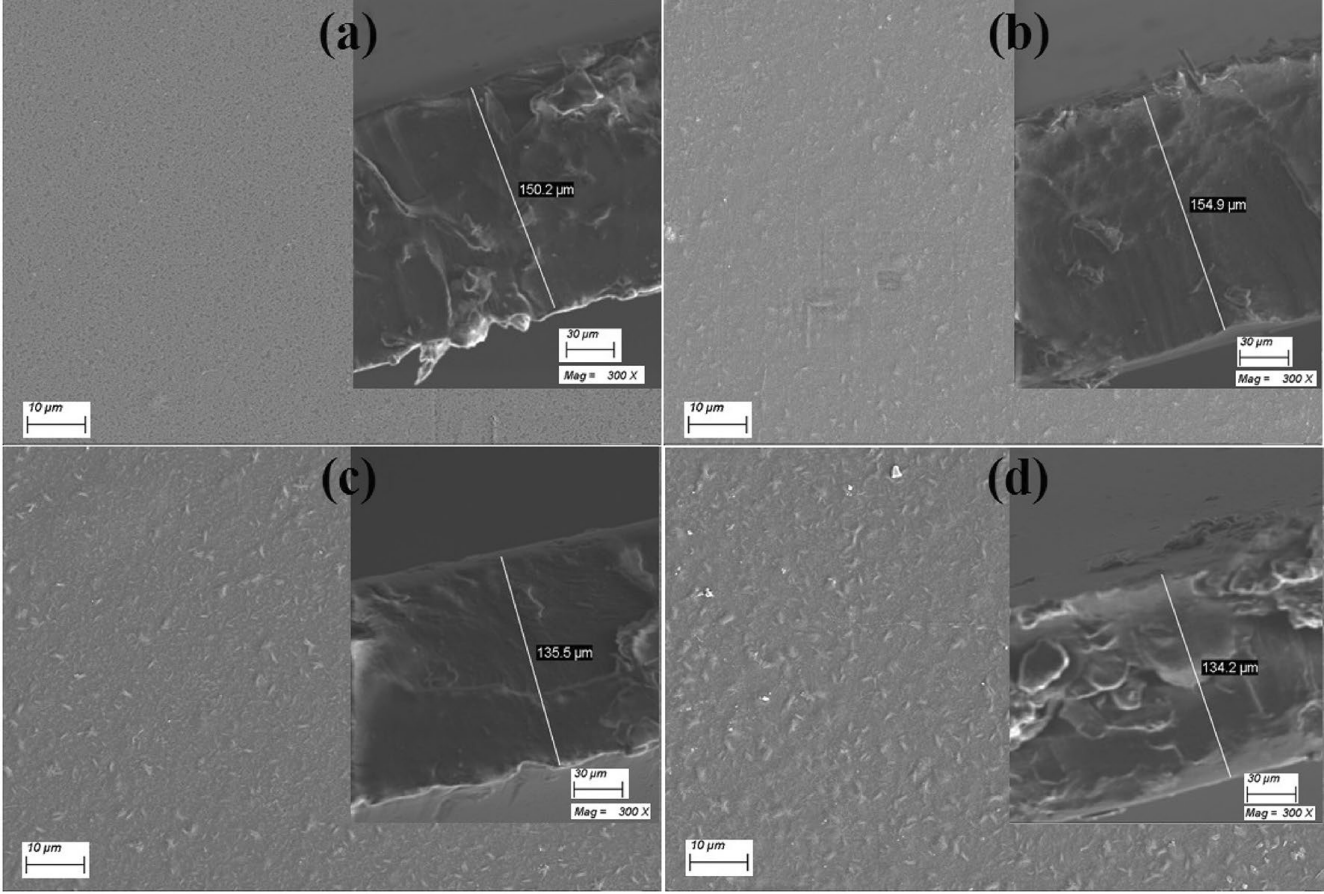
FE-SEM surface images for (**a**) CMC/St, and (**b**–**d**) CMC/St loaded with 0.1–0.3 wt% /SeO_2_ NP. The insets are the cross-sectional investigation and film thickness evaluation.

The morphology of SeO_2_ NP Was investigated using HR-TEM, as shown in Fig. 2. The SeO_2_ nanopowder is composed of NP of spherical shapes having diameters in the range of 10.52–23.44 nm, with an average particle size of 16.88 nm. The inset of Fig. 2 displays the selected area electron diffraction (SAED) of a single particle. The white rings are the result of electron diffraction, which verifies the prepared SeO_2_ NP’s good crystallinity. These results are consistent with the XRD results. FE-SEM images for the film surface are shown in Fig. 3. The insets of this figure show the cross-sectional view taken for film thickness determination. All films are nonporous, crack-free, and have a thickness (*d*) in the range of 134–150 µm, as listed in Table 1. The CMC/St blend appears homogenous and clear (Fig. 3a). This indicates the CMC and St are mixed and harmonious together, which suggests a good ordering structure. Figure 3b–d shows the uniform distribution of SeO_2_ NP on the film surface and dispersion within the blend. Increasing the SeO_2_ level to 0.3 wt% SeO_2_ results in the formation of small clusters on the film surface. Due to the high surface energy of NP, the SeO_2_ particles tend to agglomerate, although they are thickly coated with the CMC/St chains. This result illustrates that the SeO_2_ NP are strongly adhered to the CMC/St matrix, which in turn influences the physical properties of the blend. Fig. S1 shows the EDAX spectra of the CMC/St pure blend and that loaded with 0.3 wt% SeO_2_ NP. The films are composed of oxygen (*K*_α1_ at 0.27 keV) and carbon (*K*_α1_ at 0.53 keV). The film loaded with 0.3 wt*%* SeO_2_ exhibits two small peaks at 1.37–1.42 keV, representing the *L*_α_ and *L*_β_ lines of Se. In addition, the inset of Fig. S1 confirms the uniform distribution of Se atoms.

**Table 1:** Band gap (*E*_g_), the average excitation energy (*E*_o_), the dispersion energy (*E*_d_), and the ratio of carriers’ concentration to the electron effective mass (N/m*) of CMC/St/SeO_2_.

Sample	*E*_g_ (eV)		*E*_o_ (eV)	*E*_d_ (eV)	(N/m*) × 10^56^/kg/m^3^
	Direct	Indirect			
Pure CMC/St	5.50	4.8	5.07	10.55	0.318
0.1 wt% SeO_2_	5.40	4.6	4.23	11.44	0.972
0.2 wt% SeO_2_	5.10	4.3	4.76	18.31	0.986
0.3 wt% SeO_2_	5.25	4.5	5.097	25.48	1.021

**Figure 4:**
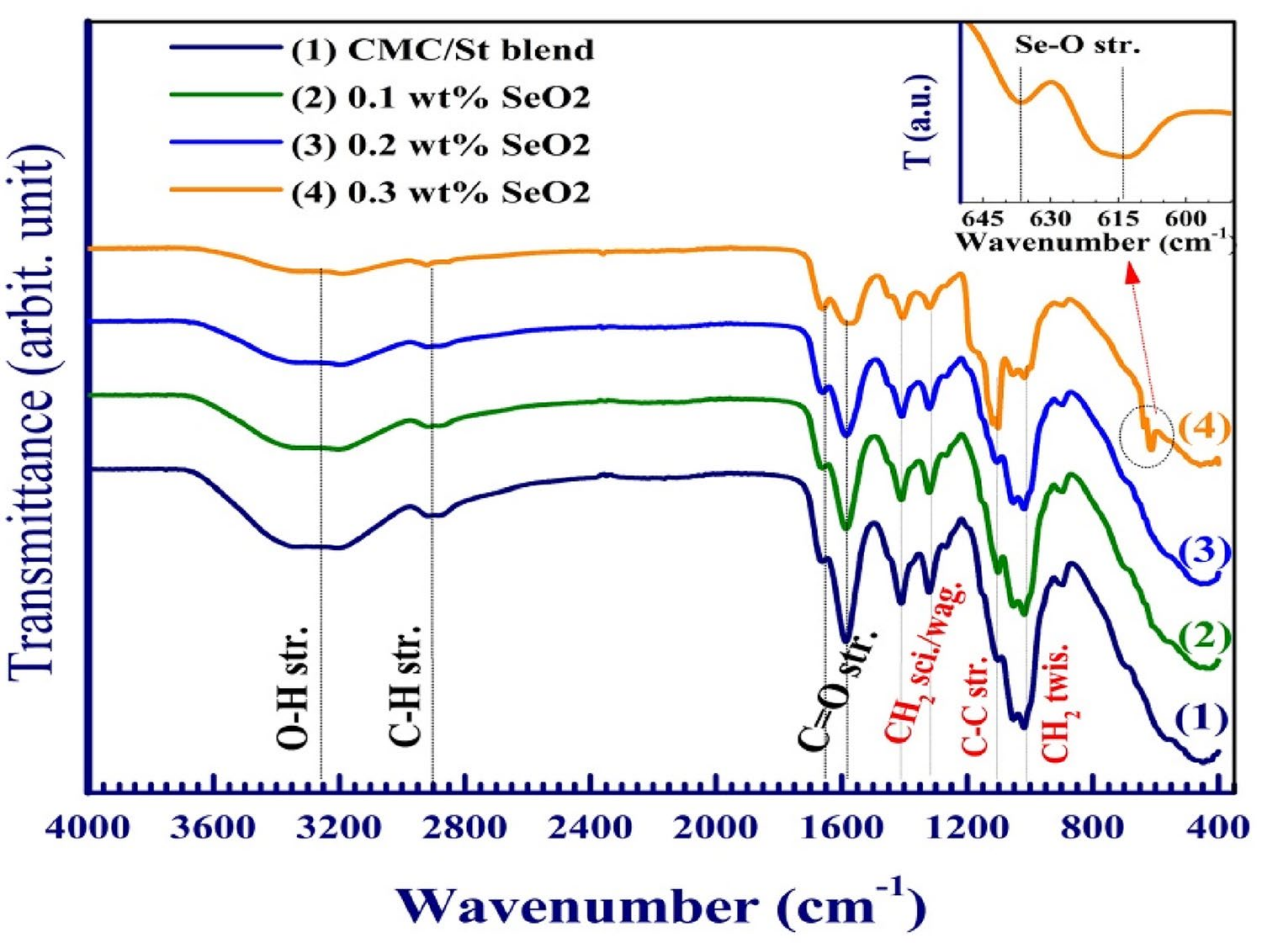
Transmission spectra (FTIR) for CMC/St, and CMC/(0.1–0.3 wt%) SeO_2_ NP.

This paragraph is devoted to exploring the existing functional groups in the films and discussing their vibrational modes through analyzing the FTIR spectra shown in Fig. 4. The broad absorption band centered around 3250 cm^−1^ is assigned to OH stretching, where both CMC and St have many OH groups. The asymmetric stretching vibration of CH_2_ is found at about 2905 cm^−1^. The relatively small band at 1655 cm^−1^ and the sharp one at 1595 cm^−1^ are assigned to the stretching vibration of the C=O groups of St and CMC (${\text{COO}}^{ - }$ groups)^4^. The CH_2_ scissoring, wagging, and twisting modes in the CMC/St blend take place at 1410 cm^−1^, 1320 cm^−1^, and 1021 cm^−1^, respectively^27^. The tiny peak in the blend spectrum at 1095 cm^−1^ may be assigned to the C─C stretching mode^28^. The stretching vibration mode of the –CH_2_–OH (primary alcoholic) is observed as a deep and sharp band at 1056 cm^−1^^8^. Loading 0.1–0.3 wt% SeO_2_ NP results in a reduction in the intensity of all bands except the one at 1095 cm^−1^, due to the hydrogen bond formed between SeO_2_ NP and the functional groups in the blend. The interactions between CMC, St, SeO_2_ NP and the hydrogen bonds formed are proposed in Fig. S2. The CMC/St blend has a lot of OH groups, which make it easier for molecules to interact with each other and for hydrogen bonds to form between the blend chains and the added NP. The spectrum of the CMC/St blend loaded with 0.3 wt% SeO_2_ has two additional adjacent small bands, as indicated in the inset of this figure, which appear at 636 and 614 cm^−1^ which could be attributed to Se–O bond vibration^26^. We expect that the changes in structure, hydrogen bonds, and complexation with the added NP will make the blend’s physical (electrical and optical) properties better^28^. This will make it useful for a wide range of practical and technological uses.

### UV–vis spectroscopy and the optical constants

**Figure 5:**
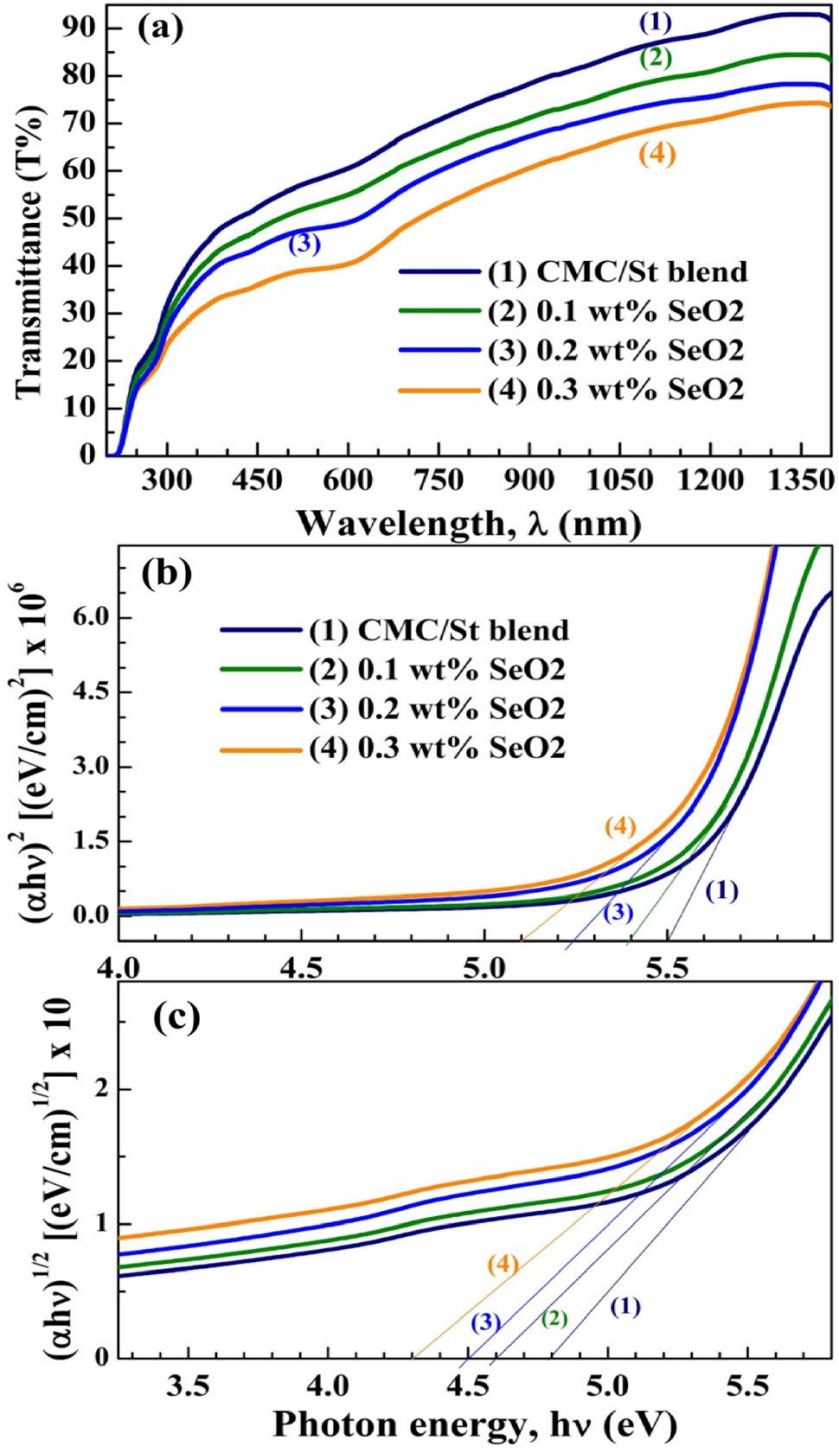
UV–vis optical properties; (**a**) transmittance spectra, (**b**) direct and (**c**) indirect band gap.

The spectra of transmittance (T%) and reflectance (R%) in the UV–vis-NIR were recorded and used to figure out the changes in the band gap structure and optical constants after adding the SeO_2_ NP in the CMC/St blend. Figure 5a displays T% of the films. In visible and IR regions, the blend and 0.3 wt% SeO_2_/blend exhibit 50–90% and 33–74% transmittance, respectively. These ranges of T% make these films suitable for optoelectronic applications. El Miri et al.^14^ reported T% between 80 and 95% in the visible light for CMC/St modified with cellulose nanocrystals. The drop in T% as the content of SeO_2_ increased is attributed to the sphere-like morphology of SeO_2_ NP, seen in Fig. 2. These NP scatter photons that hit them. The T% data were utilized for calculating the absorption coefficient (*α*) using the equation: $\alpha = \frac{1}{d}\ln \left( \frac{1}{T} \right)$. Tauc’s relations: $(\alpha h\upsilon )^{2} = A\left( {h\nu - E_{{\text{g}}}^{{\text{d}}} } \right)$ and $(\alpha h\upsilon )^{1/2} = B\left( {h\nu - E_{{\text{g}}}^{{\text{i}}} } \right)$, where *A* and *B* are constants, and *hv* is the incident photon energy, $hv \left( {{\text{eV}}} \right) = \frac{1242 }{{\lambda \left( {nm} \right)}}$, can be used to calculate the direct ($E_{{\text{g}}}^{{\text{d}}}$) and indirect $(E_{{\text{g}}}^{{\text{i}}}$) band gaps of the samples^16,23^. Figure 5b, c shows the curves of $\left( {\alpha h\upsilon } \right)^{2}$ versus *hυ* and $(\alpha h\upsilon )^{1/2}$ versus *hυ*. Extrapolating the linear portions of these curves to *α* = 0, gives the values of $E_{{\text{g}}}^{{\text{d}}}$ and $E_{{\text{g}}}^{{\text{i}}}$, which are listed in Table 1. The $E_{{\text{g}}}^{{\text{d}}}$ and $E_{{\text{g}}}^{{\text{i}}}$ of the St/Cs blend are 5.5 and 4.8 eV, respectively, shrank to 5.1 and 4.3 eV, respectively, with increasing the SeO_2_ NP content to 0.3 wt%. Similarly, the $E_{{\text{g}}}^{{\text{d}}}$ and $E_{{\text{g}}}^{{\text{i}}}$ of chitosan (70%)/PAAm (30%) were reduced from 5.32 and 5.03 eV to 5.2 and 4.83 eV, respectively, after loading 2.0 wt% Se NP^23^. In addition, the $E_{{\text{g}}}^{{\text{i}}}$ and $E_{{\text{g}}}^{{\text{d}}}$ of PVA/CMC were decreased from 4.0 and 4.84 eV to 2.18 and 2.96 eV, respectively, after loading 0.8 wt% SeO_2_^3^. Moreover, the $E_{{\text{g}}}^{{\text{d}}}$ value chitosan/St decreased to 2.23 eV after doping with Sb_2_S_3_-CeO_2_ NP^10^. Hamza and Habeeb reported T% for PVA/CMC blend loaded with 8 wt% Cr_2_O_3_–SiO_2_ NP in the range of 20–50%, and $E_{{\text{g}}}^{{\text{i}}}$ in the range of 4.26–2.91 eV^29^. It’s possible for defects to form and for charge transfer complexes and localized states to exist around the blend’s valence and conduction bands because of the SeO_2_ NP.

**Figure 6:**
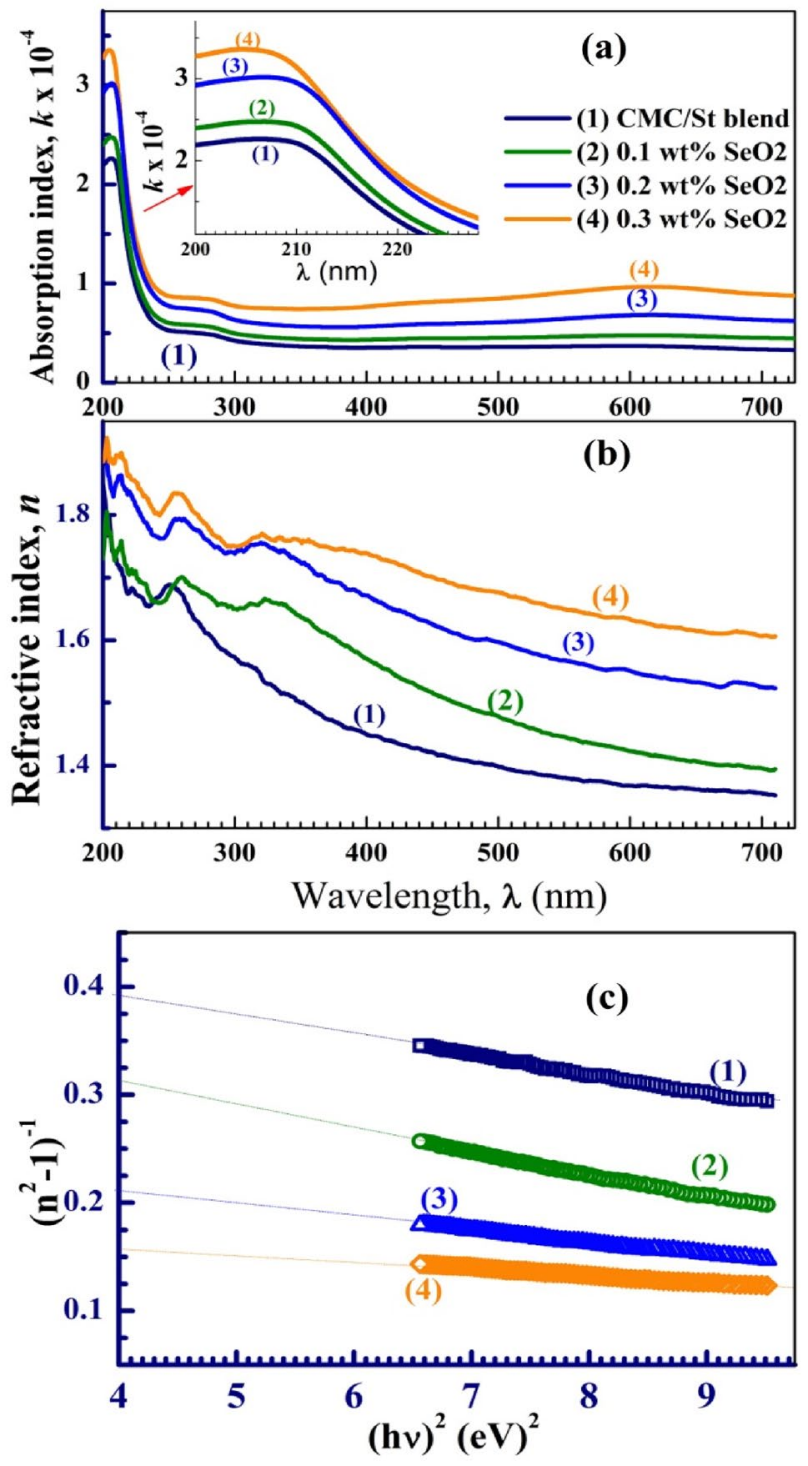
Optical constants of CMC/St/SeO_2_ nanocomposites; (**a**) absorption index (*k*), (**b**) refractive index (*n*), (**c**) determination of *E*_d_ and *E*_o_.

The absorption index ($k = \frac{\alpha \lambda }{{4\pi }}$) spectra of the blend and nanocomposites are shown in Fig. 6a. The films exhibit very low *k* values, where *k *˂ 3.5 × 10^−4^ in the UV region and k ˂ 1 × 10^−4^ in the visible and IR regions. Besides a slight absorption band at 275 nm, a clear absorption edge appears at about 208 nm for all samples; see the inset of this figure. These bands arise due to π → π* (C=C/C=O) and n → π* (C–O/C–H) electronic transitions. The noticeable intensity change of these bands between the CMC/St and CMC/St/SeO_2_ may be owing to the increase in disorder and amorphous regions caused by SeO_2_ NP loaded inside the host matrix^30,31^. Moreover, the 0.2 and 0.3 wt% SeO_2_-loaded films display a hump around 600 nm that could be assigned to the formation of charge transfer complexes. Similar observations were found in the absorption spectrum of PVA/ Cr_2_O_3_^32^ and in the spectrum of Cs/PAAm/0.4 wt% Se nanocomposites^24^.

Evaluating the optical constants for the samples, such as the refractive index (*n*), where $$n = \frac{speed of light in the sample}{{3{\text{ x}} 10^{8} m/s}}$$, is essential for optical communications and device fabrication. The obtained reflectance (R%) spectra were used to calculate the *n* values of the films using the equation: $$n = \frac{1 + \sqrt R }{{1 - \sqrt R }}$$^33^). The distribution of *n* with *λ* is shown in Fig. 6b. In the studied range of *λ*, 2 *˂ n ˂ *1.3, where the values of *n* take a wave-like behavior in the UV region and decrease with λ in the visible region of the spectrum. The improved reflectivity of the material after the incorporation of SeO_2_ NP inside the blend is due to the scattering effect of the dispersed NP. This enhancement in *n* values makes CMC/St/SeO_2_ composites suitable for some top-end advanced optical and electronic equipment such as anti-reflective coatings, waveguides, and light-emitting diodes.

**Figure 7:**
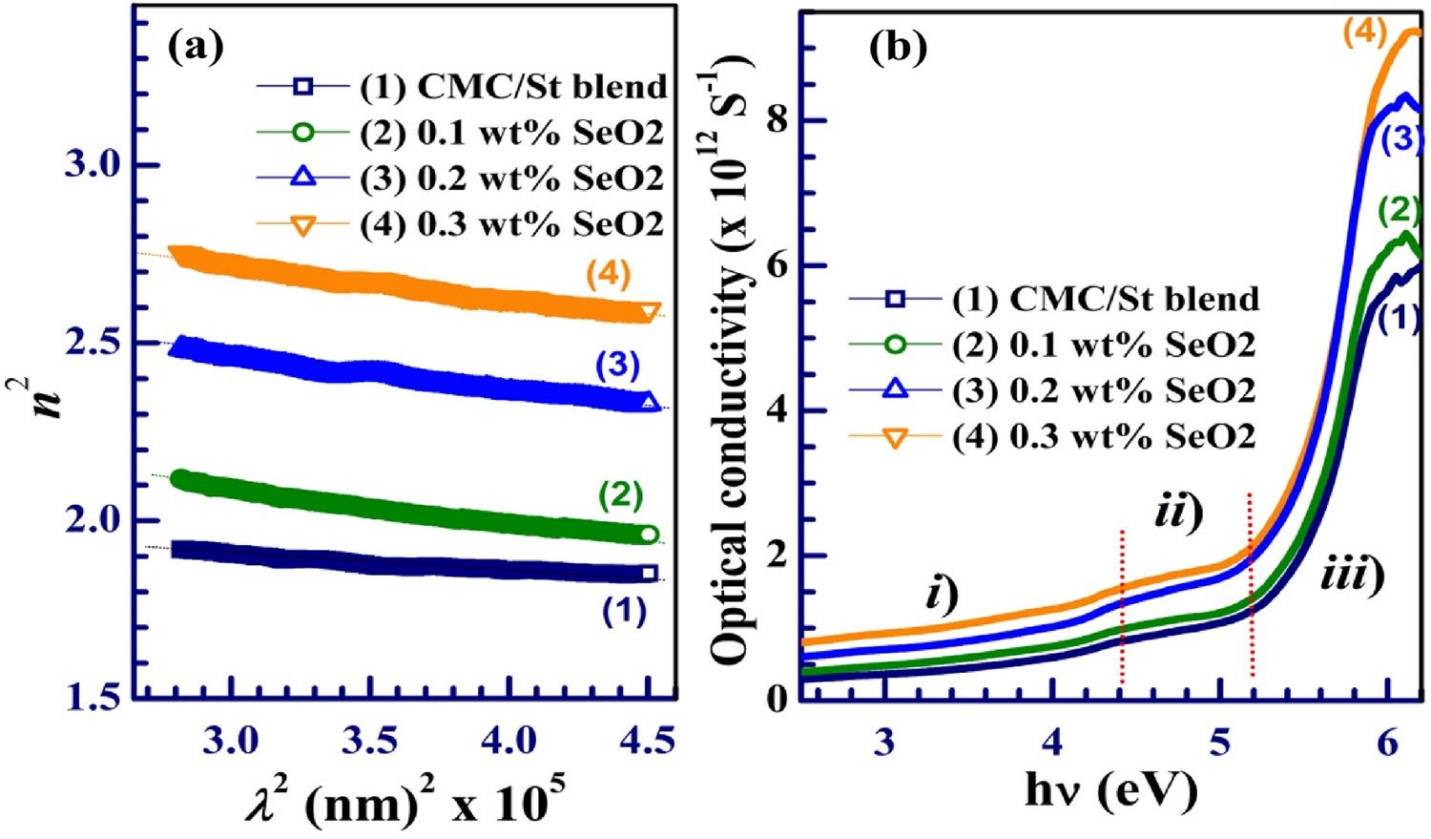
(**a**) N/m* determination, and (**b**) optical conductivity versus the photon energy of CMC/St/SeO_2_ nanocomposites.

The average excitation energy (*E*_o_) for the electronic transitions, the dispersion energy *E*_d_, the carriers’ concentration divided by the electron effective mass (N/m*), and the optical conductivity ($\sigma_{op}$) are other optical constants important for designing the optoelectronic components and devices. These parameters can be evaluated by looking at how *n* decreases with λ (Fig. 7b) and how well they fit the Wemple and Di-Domenico theory. They can also be found using the following relationships^34–36^:

1 $$ (n^{2} - 1) = \frac{{E_{d} E_{o} }}{{E_{o}^{2} - (h\nu )^{2} }} $$

2 $$ n^{2} = \varepsilon_{l} - \lambda^{2} \left( {\frac{{e^{2} }}{{\pi c^{2} }}} \right)\left( {\frac{N}{{m^{*} }}} \right) $$

3 $$ \sigma_{op} = \frac{\alpha nc}{{4\pi }} $$

where *ε*_*L*_, *e*, and *c* are the lattice dielectric constant, electron charge, and velocity of light, respectively. the *E*_o_ and *E*_d_ were obtained from the intercepts and slopes of the linear portions of the (*n*^2^*–*1)^−1^ versus (*hν*)^2^ plots, as shown in Fig. 6c. The N/m* values were determined from the slope of Fig. 7a, and the obtained values are listed in Table 1. The gradual increase in N/m* values from 0.318 × 10^56^/kg/m^3^ for the pure blend to 1.021 × 10^56^/kg/m^3^ at 0.3 wt% SeO_2_ NP loading is consistent with the observed narrowing in the *E*_g_. This means improving the semiconducting nature of the CMC/St blend with SeO_2_ NP incorporation. Figure 7b shows the dependence of $\sigma_{op}$ on *hυ*. To explain the observed behavior of $\sigma_{op}$, the curves can be divided into three regions. (i) The $\sigma_{op}$ increases slightly with *hυ* till *hυ ˂ *4.4 eV, where these limited energy values can excite a small number of the charge carriers to participate in the conduction process. (ii) A plateau region (4.4 ˂ *hυ ˂ *5.2 eV), $\sigma_{op}$ values appear constant. In region (iii) the incident energy (*hυ ˃ *5.2 eV) is larger than the direct *E*_g_, so that $\sigma_{op}$ increases sharply, where the photons of the UV region have enough energy to excite the charge carriers to higher energy levels^37^. In this region, $\sigma_{op}$ of the films is in the range of 2 × 10^12^–6 × 10^12^ S^−1^, which is larger than the values of 0.95 × 10^12^ –1.68 × 10^12^ S^−1^ reported for PVA/CMC blend loaded with Cr_2_O_3_–SiO_2_ NP up to 8.0 wt%^29^. Moreover, $\sigma_{op}$ of CMC was found to be less than 2.0 × 10^11^ S^−1^ increased to be in the range of 4.0 × 10^12^ S^−1^–6.0 × 10^1^ S^−1^ after blending with polypyrrole^2^. This means that St greatly improves the $\sigma_{op}$ of CMC. At *hυ ˃ *6.0 eV a saturation state is reached. SeO_2_ NP loading induced structural changes, defect states, and charge transfer complex formation, as discussed in the previous sections. Therefore, a significant improvement in $\sigma_{op}$ with increasing SeO_2_ NP content is noticed. The changes that SeO_2_ NP caused in the optical parameters show that they are effective for improving the optical properties of the CMC/St blend. These enhancements make these compositions suitable for optoelectronic devices like photovoltaic cells and organic light-emitting diodes^38^.

### *I*–*V Characteristics, conductivity, and conduction mechanism*

**Figure 8:**
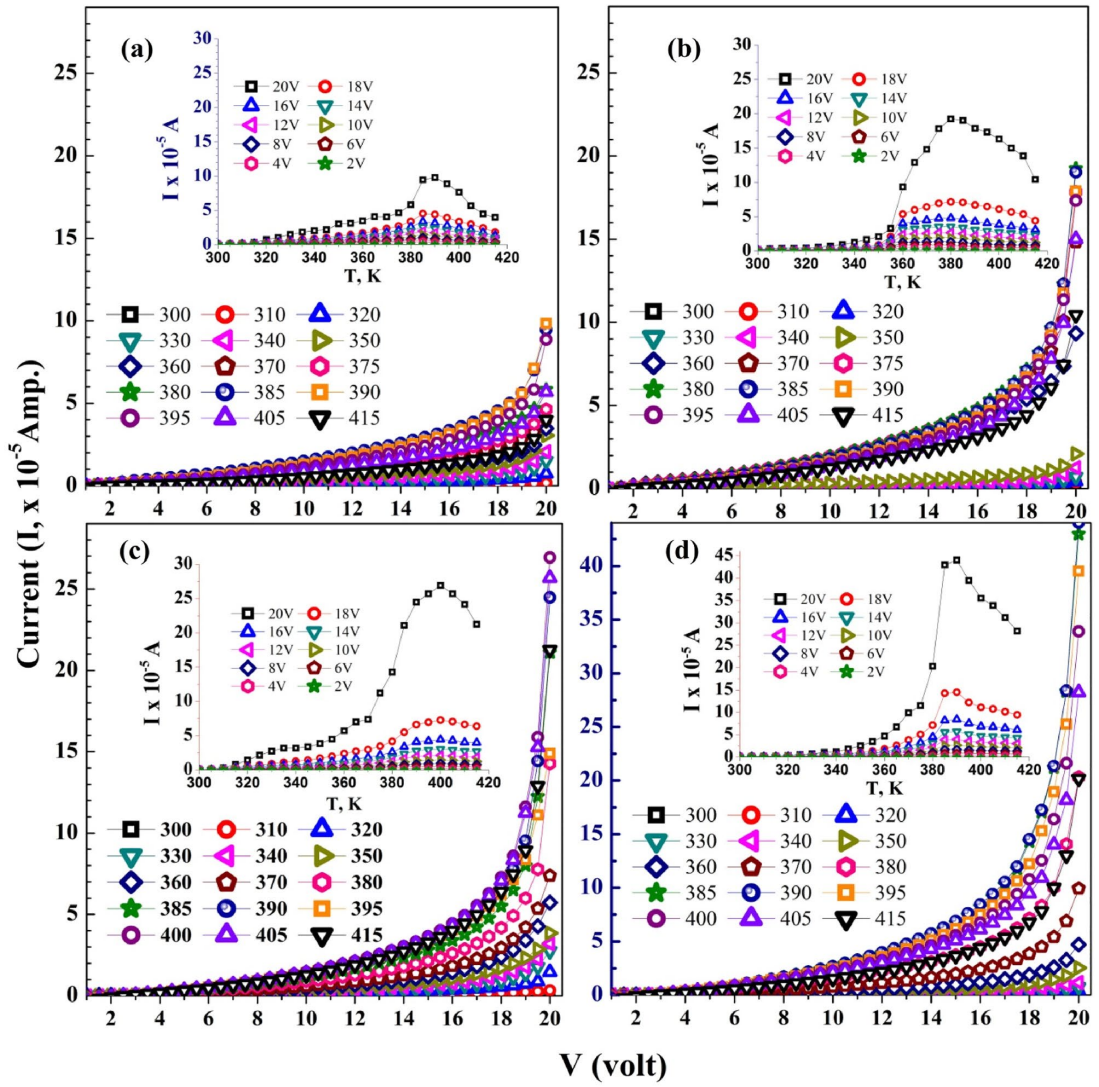
I–V curves in the temperature range of 300–415 K for (**a**) the blend, and (**b**–**d**) the blend loaded with 0.1–0.3 wt% SeO_2_ NP. The insets show the variation of *I* with T at 2.0–20 V.

The *I–V* characteristic curves of the CMC/St blend and its nanocomposites are shown in Fig. 8a–d. In the applied volts range of 0.5–20 V, the obtained *I* is increased with increasing the temperatures to a certain degree: 390 K for blend, 0.1 and 0.3 wt% SeO_2_ NP-loaded films, and 400 K at 0.2 wt% SeO_2_ content. After this limit, *I* decreased with increasing temperature. This behavior indicates that the temperature has a decisive effect on *I*, whatever the applied voltage. All films permit a current in the order of 10^−5^ A, but the SeO_2_ NP-loaded films have a higher *I*. The relation: $I = F V^{r}$^39^, where *F* is a constant, can be used to determine the nonlinear coefficient parameter (*r*) for reporting the conduction mechanism in polymers^40^. When *r* = 1, then the ohmic behavior is dominant. A value of *r* = 2 means that trap-free-space-charge-limited is the leading mechanism. For *r* > 2, the space-charge-limited mechanism could be a suitable conduction mechanism^41^. The *r* values are derived from the slope on the linear portions of Ln (*I*)–Ln(*V*) curves, as depicted in Fig. S3 and listed in Table S1. As noticed, *r* values are in the range of 1.06–195, 1.45–1.95, 1.63–2.14, and 2.07–2.23 for the blend pure and loaded with 0.1, 0.2, and 0.3 wt% SeO_2_ NP, respectively. These values confirm the non-ohmic feature of the *I–V* characteristics of the materials under study. Increasing *r* after doping implies that the traps become larger or deeper.

**Figure 9:**
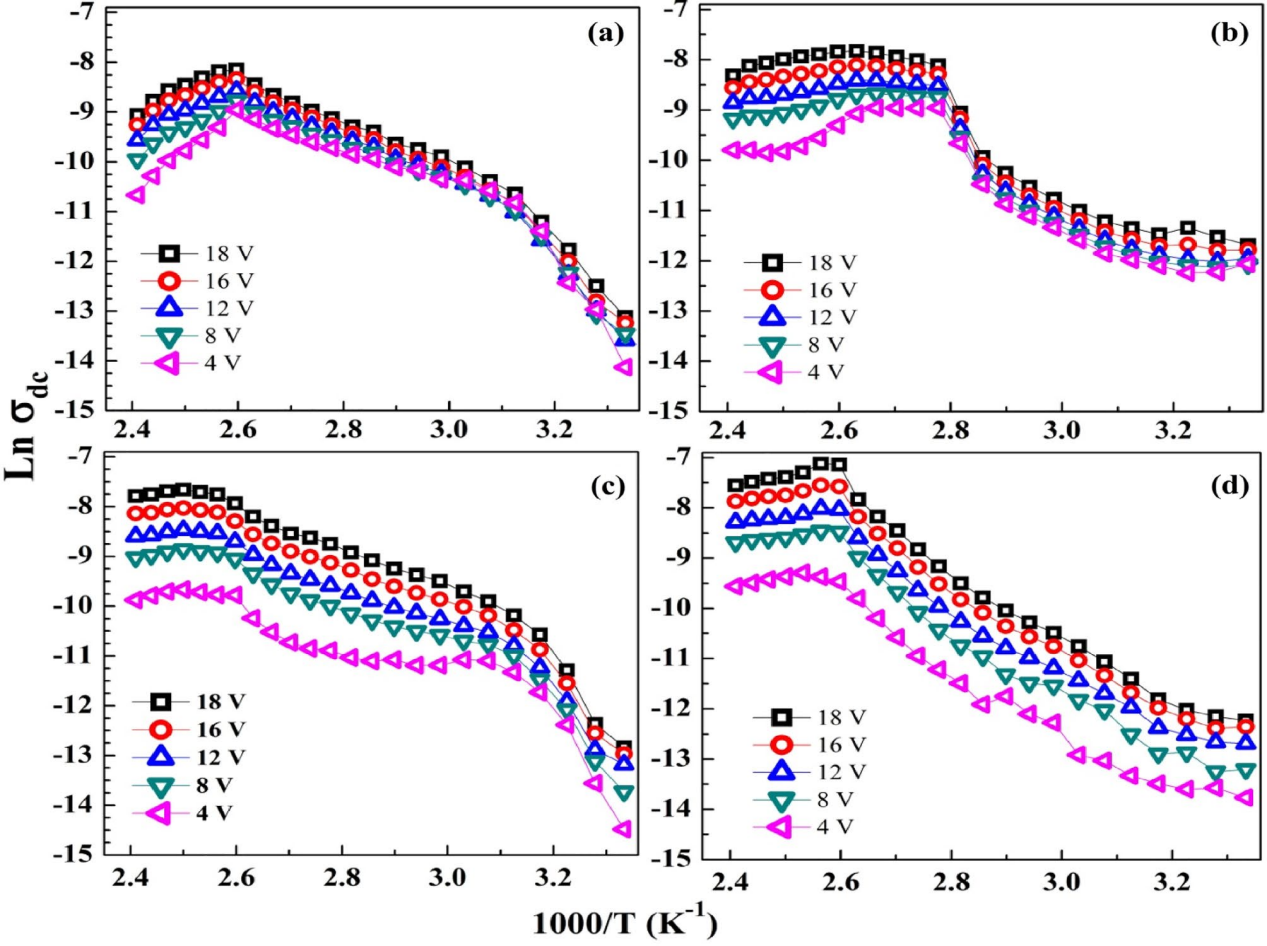
Variation of Ln(σ_dc_) with (1000/T) for (**a**) CMC/St blend and (**b**–**d**) blend loaded with 0.1–0.3 wt% SeO_2_ NP.

Figure 9a–d shows the dependence of dc conductivity ($\sigma_{dc}$) on the temperatures as Ln ($\sigma_{dc} )$versus (1000/T), verifying the Arrhenius relation^42^:

4 $$ \sigma_{dc} = \sigma_{0} {\text{exp}}\left( {\frac{{E_{a} }}{{k_{B} T}}} \right), $$

where $\sigma_{0}$ is the conductivity at infinite temperature, *k*_B_ is the Boltzmann constant, and $E_{a}$ is the activation energy. The $\sigma_{DC}$ of the investigated films was calculated by using the relation; $\sigma_{dc} = \frac{I.d}{{V.A}}$, where *d* and *A* are the sample thickness and cross-sectional area, respectively. Some notes can be drawn from this figure: (i) The $\sigma_{dc}$ of the blend varies between 3.8 × 10^−7^ to 5.6 × 10^−4^ S/m, according to the applied temperature. These values are consistent with the published results^39^. (ii) The overall temperature dependence of the $\sigma_{dc}$ curves can be divided into three distinct regions: At low (300–320 K) and moderate (320–380 K) temperatures, the Arrhenius behavior is verified with a high rate of $\sigma_{dc}$ improvement at low temperatures due to the available thermal activation of the blend chains in this region, followed by a relatively lower rate of $\sigma_{dc}$ improvement in the moderate region of temperatures. In the third region or high temperatures (380–415 K), a decrement trend in $\sigma_{dc}$ is observed. The blend loaded with 0.2 wt% SeO_2_ NP exhibits similar behavior to that of the blend but with higher and constant $\sigma_{dc}$ at higher temperatures (385–415 K). The $\sigma_{dc}$ curves of the 0.1 and 0.3 wt% SeO_2_ NP blend are divided into two regions only; the first one extends in the regions of 300–360 K and 300–380 K, where $\sigma_{dc}$ increases with temperature. The second one is at temperatures in the range of 360–415 K for 0.1 wt% SeO_2_ and 380–415 K for 0.3 wt% SeO_2_ content, where $\sigma_{DC}$ decreases at a low rate with temperature. At the higher side of temperatures, $\sigma_{DC}$ of 0.3 wt% SeO_2_/blend ˃ $\sigma_{dc}$ of 0.1 wt% SeO_2_ blend ˃ $\sigma_{dc}$ of the blend. This means increasing SeO_2_ NP content results in raising $\sigma_{dc}$ of the blend. This result is consistent with the XRD and UV/vis data, where the reduced crystallinity and increasing disorder made the *E*_g_ of the blend more shrinkable with an increasing SeO_2_ NP ratio. The uniform distribution of SeO_2_ NP facilitates the formation of a continuous network throughout the blend matrix.

To shed more light on the conduction mechanism, we will consider the following relations between the current density $\left( {J = \frac{I}{A}} \right)$ and the applied electric field $\left( {E = \frac{V}{d}} \right)$^4,43,44^: for the Schottky (Sc) mechanism:

5 $$ J = GT^{2} \exp \left( { - \frac{\varphi }{{k_{B} T}}} \right){\text{exp}}\left( {\frac{{\beta \left( {Sc} \right)E^{0.5} }}{{k_{B} T}}} \right), $$

and Poole–Frenkel (PF) mechanism:

6 $$ J = \frac{{\sigma_{o} V}}{d}{\text{exp}}\left( {\frac{{\beta \left( {PF} \right)E^{0.5} }}{{k_{B} T}}} \right) $$

where T is the absolute temperature, *k*_B_ = 1.379 × 10^−23^ J/K (Boltzmann constant), *G*, and* φ* are constants, and $\sigma_{o}$ is the low field conductivity. The Sc emission is related to the barrier at the surface of a metal or insulator, whereas the PF emission is related to the barrier in the bulk of the material. Theoretically, it was concluded that:

7 $$ {\upbeta }\left( {{\text{Sc}}} \right) = \sqrt {\frac{e}{{4\pi \varepsilon_{o} \varepsilon^{\prime}}}} \;{\text{and}}\;{\upbeta }\left( {{\text{PF}}} \right) = \sqrt {\frac{e}{{\pi \varepsilon_{o} \varepsilon^{\prime}}}} , $$

where $\varepsilon_{o}$ is the permittivity of free space and $\varepsilon^{\prime}$ is the permittivity of the material. This means that ${\upbeta }\left( {{\text{PF}}} \right)$ = 2 ${\upbeta }\left( {{\text{Sc}}} \right)$. For our blend, we can take $\varepsilon^{\prime}$ = 5^45^, the ${\upbeta }\left( {{\text{Sc}}} \right){ }$ and ${\upbeta }\left( {{\text{PF}}} \right){ }$ should be in the order of 1.69 × 10^−5^ and 3.39 × 10^−5^, respectively.

**Figure 10:**
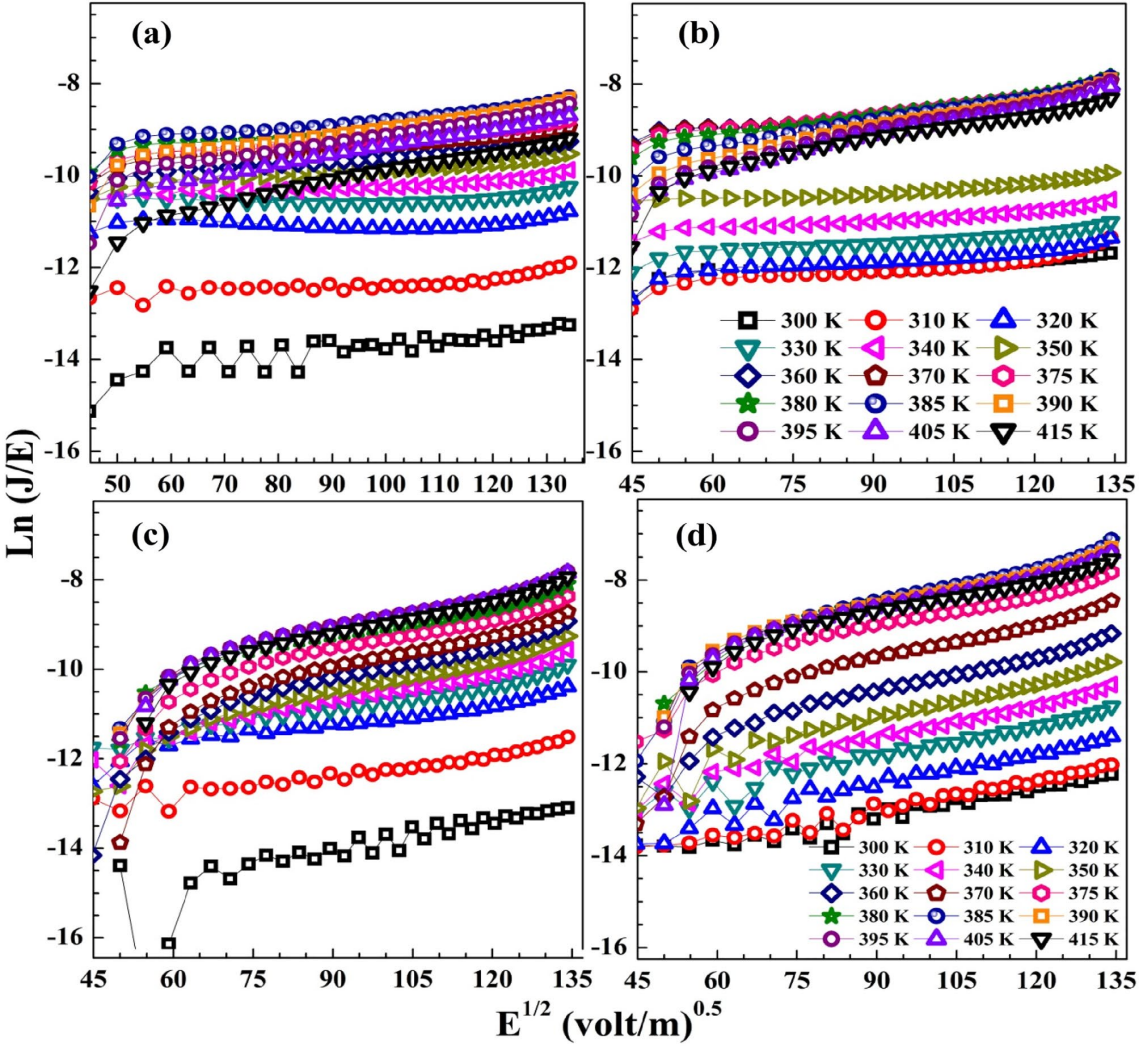
Ln(J/E) versus E^0.5^ to determine the conduction mechanism in (**a**) CMC/St blend and (**b**–**d**) the blend loaded with 0.1–0.3 wt% SeO_2_ NP.

Fig. S4 and Fig. 10a–d display the curves of Ln (J) versus *E*^0.5^ and Ln (*J/E*) versus *E*^0.5^, respectively, for the blend and its nanocomposites. The $\beta \left( {{\text{Sc}}} \right)$ and $\beta \left( {{\text{PF}}} \right)$ values were determined on the slopes of Fig. S4 and Fig. 10a–d, respectively, as $\beta$ = slope × *k*_B_T, and the values are listed in Table 2. As noticed, the values of $\beta$ are consistent with the theoretical value of ${\upbeta }\left( {{\text{PF}}} \right)$, thus the PF emission is the most suitable conduction mechanism in CMC/St blend, and their composites with 0.1–0.3 wt% SeO_2_ NP. The conduction mechanism in the CMC /PVP (75%/25%) blend and NiO/CMC/PVP nanocomposites was found to be PF emission^4^, while in the CMC/PVA (80%/20%) blend it was found to be Sc emission. However, doping with CuO NP into this blend matrix and heating led to the PF emission^25^. Therefore, the mechanism of conduction in polymeric and nanocomposite materials depends on the blend structure, temperature, and nanofillers.

**Table 2:** $\beta \left( {{\text{PF}}} \right)$ and $\beta \left( {{\text{Sc}}} \right)$ values derived from the slopes of Fig. 10 and Fig. S4.

Sample	$\beta$ value	at			
		300 K	310 K	330 K	350 K
CMC/St blend	$\beta \left( {{\text{PF}}} \right)$	3.68 × 10^−5^	8.14 × 10^−5^	1.89 × 10^−5^	6.60 × 10^−5^
	$\beta \left( {{\text{Sc}}} \right)$	1.64 × 10^−4^	1.27 × 10^−4^	1.31 × 10^−4^	1.68 × 10^−4^
0.1 wt% SeO_2_ NP	$\beta \left( {{\text{PF}}} \right)$	4.14 × 10^−5^	3.62 × 10^−5^	3.72 × 10^−5^	2.50 × 10^−5^
	$\beta \left( {{\text{Sc}}} \right)$	1.69 × 10^−4^	1.5 × 10^−4^	1.5 × 10^−4^	1.34 × 10^−4^
0.2 wt% SeO_2_ NP	$\beta \left( {{\text{PF}}} \right)$	1.37 × 10^−5^	8.98 × 10^−5^	7.48 × 10^−5^	5.10 × 10^−5^
	$\beta \left( {{\text{Sc}}} \right)$	2.26 × 10^−4^	1.91 × 10^−4^	1.82 × 10^−4^	2.20 × 10^−4^
0.3 wt% SeO_2_ NP	$\beta \left( {{\text{PF}}} \right)$	5.41 × 10^−5^	6.23 × 10^−5^	6.23 × 10^−5^	5.17 × 10^−5^
	$\beta \left( {{\text{Sc}}} \right)$	2.68 × 10^−4^	2.44 × 10^−4^	2.36 × 10^−4^	2.26 × 10^−4^

## Conclusion

Well-crystallized SeO_2_ NP was solvothermally prepared and loaded within the CMC/St bio-blend. Increasing the SeO_2_ level from 0.1 to 0.3 wt% increased the amorphous regions inside the blend. SeO_2_ NP with a spherical shape and a crystallite size of 16.88 nm was spread out evenly on the surface of the film and worked well with the functional groups of the blend. At 0.3 wt% SeO_2,_ the transmission of the blend decreased from 50–90% to 33–74%, and the direct and indirect band gaps decreased from 5.5 and 4.8 eV to 5.25 and 4.5 eV, respectively. The dispersion optical parameters *E*_o_, *E*_d_, N/m*, and optical conductivity were improved with increasing SeO_2_ NP content. All samples exhibited non-ohmic features based on the *I-V* characteristics. The $\sigma_{dc}$ displayed Arrhenius behavior. Increasing the amorphous regions and defects inside the blend and shrinking the band gap and the continuous network formed by SeO_2_ NP throughout the blend matrix led to the observed increase in $\sigma_{dc}$. Based on the dependence of *J* on *E* in the temperature range 300–415 K (Ln (*J/E*) vs. *E*^0.5^), Poole–Frenkel emission is found to be the most favorable conduction mechanism in the nanocomposite films. In summary, SeO_2_ NP led to structural changes inside the CMC/St blend and improved the optical and electrical properties of the blend. Therefore, the obtained materials are best suited for coatings, optoelectronic applications, and related devices such as photovoltaic cells and organic light-emitting diodes.

### Supplementary Information

Supplementary Information.

## Data Availability

The datasets used and/or analyzed during the current study available from the corresponding authors on reasonable request.
