# Supplementary material for: Improving the structural, optical, and electrical properties of carboxymethyl cellulose/starch/selenium oxide nanocomposites for flexible electronic devices
Source: Sci Rep. 2024 Feb 10;14:3398. doi: 10.1038/s41598-024-53268-w (PMC10858174; doi:10.1038/s41598-024-53268-w)
Supplement: Supplementary file 1 — Supplementary Information. [file 41598_2024_53268_MOESM1_ESM.pdf]

Improving the structural, optical, and electrical properties of carboxymethyl cellulose/starch/selenium oxide nanocomposites for flexible electronic devices

Adel M. El Sayed[1*], Tarek I Alanazi[2]**

[1]Physics Department, Faculty of Science, Fayoum University, El-Fayoum 63514, Egypt

[2]Department of Physics, College of Science, Northern Border University, Arar 73222, Saudi Arabia

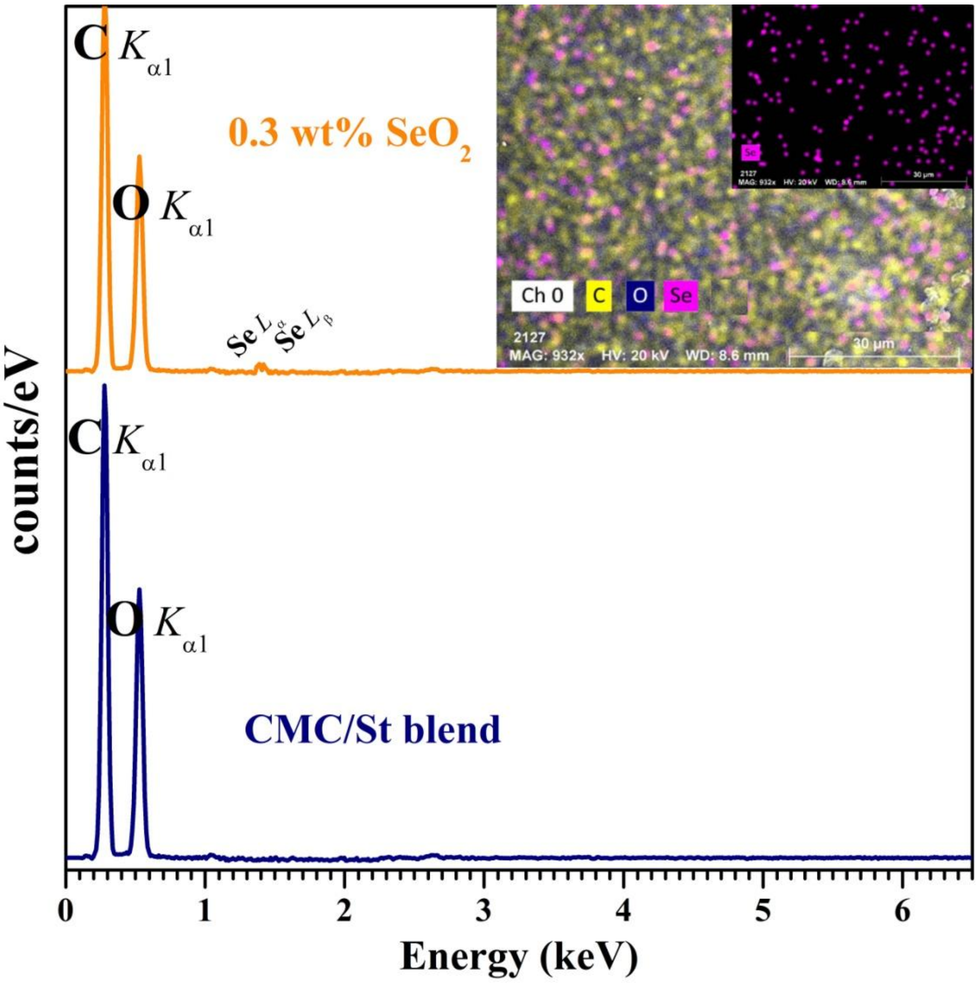

Fig. S1: EDAX spectra of CMC/St and CMC/St/0.3 wt% SeO_2.

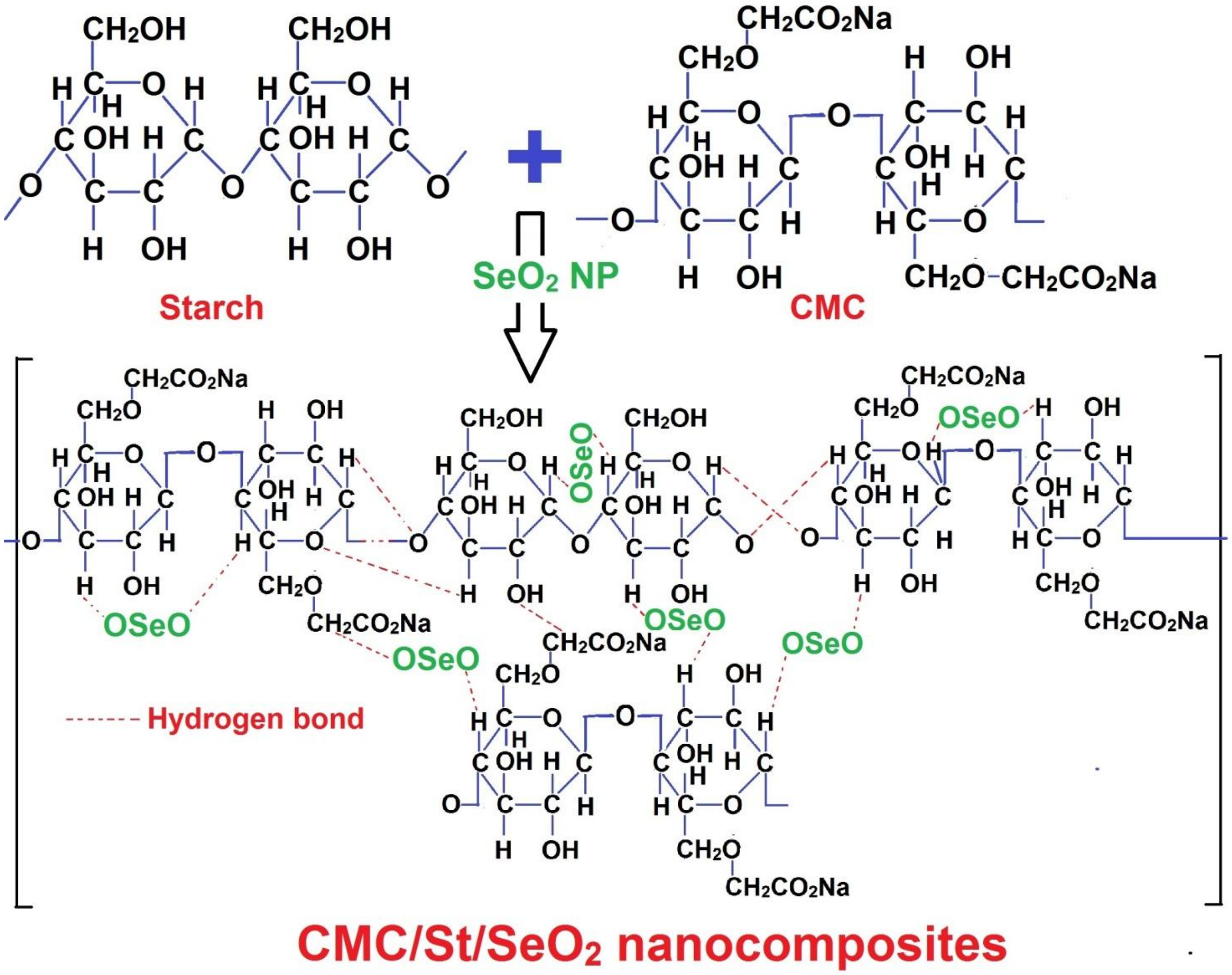

Fig. S2: Chemical structure and proposed reaction between CMC, starch and SeO_2 NP.

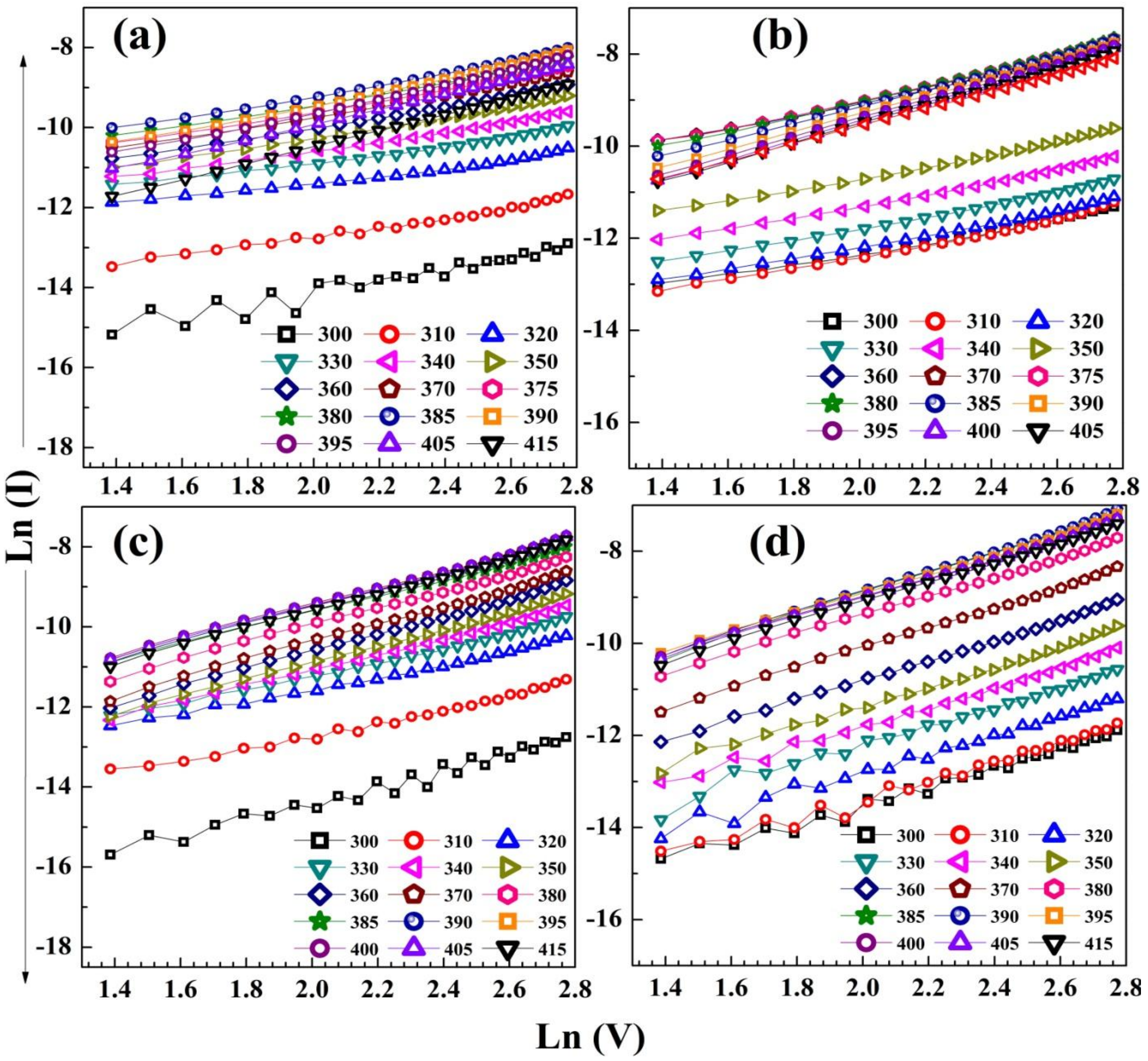

Fig. S3: Ln (***I***)–Ln (***V***) curves for (a) the blend, and (b-d) 0.1–0.3 wt% SeO_2 NP loaded films.

Table S1: Values of the nonlinear coefficient parameter (*r*)

	**300 K**	**310 K**	**320 K**	**330 K**	**350 K**	**370 K**	**390 K**	**410 K**
CMC/St blend	1.44	1.29	1.06	1.16	1.34	1.46	1.78	1.95
0.1 wt% SeO_2 NP	1.46	1.59	1.45	1.45	1.50	1.74	1.95	1.91
0.2 wt% SeO_2 NP	2.07	1.71	1.63	1.79	2.09	2.14	2.07	2.08
0.3 wt% SeO_2 NP	2.07	2.10	2.10	2.12	2.23	2.17	2.15	2.11

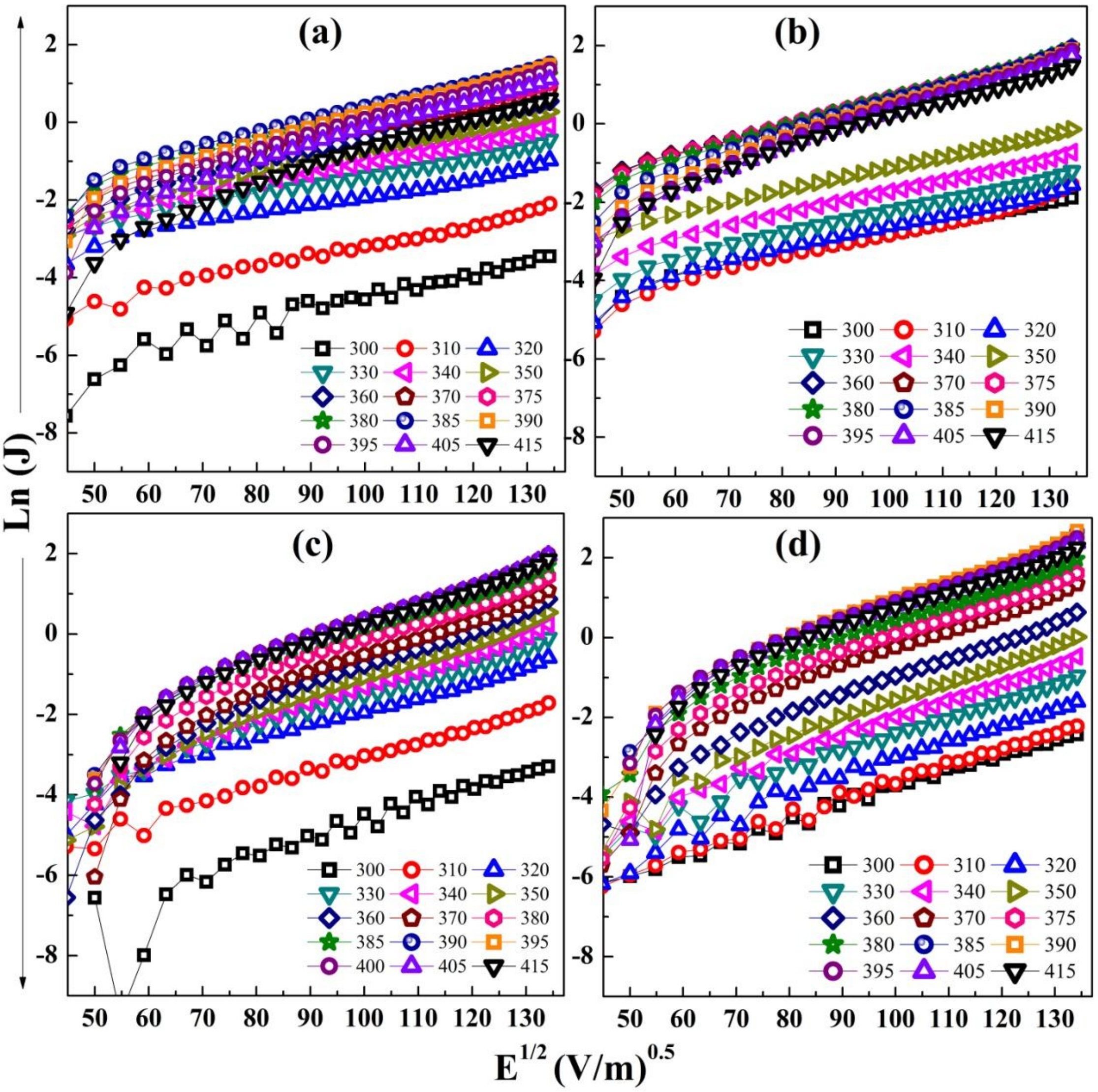

Fig. S4: Ln (J) vs $E^{1/2}$ curves for the CMC/St blend and CMC/St/SeO_2 nanocomposites.
